# Draft genome sequences of 22 *Shigella* sp. isolates recovered from human stool samples in Senegal

**DOI:** 10.1128/mra.00678-26

**Published:** 2026-07-22

**Authors:** Cheikh Ibrahima Lo, Ousmane Sadio, Elhadj Aly Niang, Adja Bousso Guèye, Safiétou Sankhe, Marie Odile Ndiaye, Moussa Moise Diagne, Yakhya Dièye

**Affiliations:** 1 Pôle Microbiologie, Institut Pasteur de Dakar, Dakar, Sénégal; 2 Plateforme de Séquençage du Pôle de Virologie, Institut Pasteur de Dakar, Dakar, Sénégal; 3 Groupe de Recherche Biotechnologies Appliquées & Bioprocédés Environnementaux (GRBA-BE), École Supérieure Polytechnique, Université Cheikh Anta Diop, Dakar, Sénégal; Rochester Institute of Technology, Rochester, New York, USA

**Keywords:** *Shigella*, genomes, Senegal, West Africa

## Abstract

*Shigella* species frequently cause bacillary dysentery and remain a major public health concern in low- and middle-income countries. We report draft genomes of 22 Senegalese *Shigella* isolates including *S. sonnei*, *S. flexneri*, and *S. boydii*. Genome sizes range from 4.34 to 5.54 Mb, with GC contents ranging from 50.25% to 50.73%. These data provide a resource for genomic epidemiology and antimicrobial resistance of *Shigella* in West Africa.

## ANNOUNCEMENT

Species of the genus *Shigella* are among the leading bacterial causes of diarrheal disease worldwide, particularly affecting young children in low- and middle-income countries (1, 2). Whole-genome sequencing (WGS) provides high-resolution insights into pathogen population structure, transmission dynamics, and antimicrobial resistance (AMR) and is increasingly used for public health surveillance (3, 4).

Despite the recognized public health importance of shigellosis, genomic data from West Africa remain limited. Recent studies from other African settings, including Ethiopia, have demonstrated the utility of WGS for characterizing circulating *Shigella* lineages and antimicrobial resistance determinants, highlighting the growing application of genomics on the continent (5). In parallel, a recent systematic review and meta-analysis underscored the high burden of *Shigella* infections and substantial antimicrobial resistance across Africa, reinforcing the need for expanded genomic surveillance efforts (6).

In this study, 22 *Shigella* isolates were recovered from stool samples of patients presenting with diarrhea at sentinel surveillance sites in Senegal between 2020 and 2021. A loopful of each stool sample was directly streaked onto *Salmonella-Shigella* (SS) agar (Oxoid Ltd., Basingstoke, UK) and Hektoen-Enteric agar (Liofilchem S.r.l., Roseto degli Abruzzi, Italy) and incubated at 37°C for 18–24 h. In parallel, a portion of each sample was inoculated into 9 mL of buffered peptone water for enrichment before subsequent subculture onto the same selective media. Presumptive isolates were identified using matrix-assisted-laser-desorption-ionization time-of-flight (MALDI-TOF) mass spectrometry (Biotyper Sirius, Bruker Daltonics, Bremen, Germany).

Bacterial genomic DNA was extracted using the QIAamp DNA Mini Kit (QIAGEN, Hilden, Germany) according to the manufacturer’s instructions. DNA concentrations were measured using a Qubit 4 Fluorometer (Thermo Fisher Scientific, Waltham, MA, USA). Sequencing libraries were prepared using the Nextera XT DNA Library Preparation Kit (Illumina, San Diego, CA, USA). Libraries were generated by tagmentation, indexed using IDT for Illumina PCR Unique Dual Indexes, purified with Agencourt AMPure XP beads, quantified, normalized, and pooled. Whole-genome sequencing was performed on an Illumina NextSeq 550 platform using a high-output 300-cycle kit, generating paired-end reads of 2 × 150 bp. Sequencing produced high coverage across all isolates, ranging from approximately 119× to 180×.

Raw reads were quality-checked using FastQC v0.11.9 (7) and trimmed using fastp v0.23.2 (8). Default parameters were used unless otherwise specified. Trimmed reads were assembled *de novo* using SPAdes v3.15.5 (9). Assembly quality was assessed using QUAST v5.2.0 (10). Genome completeness and contamination were evaluated using CheckM v1.1.3 (11), and taxonomic assignment was confirmed using Kraken2 v2.1.2 (12). Genome annotation was automatically generated by the NCBI Prokaryotic Genome Annotation Pipeline (PGAP) during genome submission to GenBank. Multilocus sequence typing (MLST) was performed using the MLST tool (v2.12.0) with the *Escherichia*/*Shigella* MLST scheme available in the PubMLST database to assign sequence types (STs).

**TABLE 1:** Genome assembly metrics of 22 *Shigella* isolates sequenced in this study

Isolate ID	Species	ST	Genome size (bp)	GC (mol%)	Coverage (×)	Number of contigs	Largest contigs	N50	L50	Assembly accession	SRA run accession
DAK01014	*Shigella sonnei*	152	4,586,069	50.48	173	542	88,193	23,353	57	GCA_056643295.1	SRR39062455
DAK02010	*Shigella sonnei*	152	4,590,191	50.50	137	575	88,199	23,265	59	GCA_056643275.1	SRR39062454
DAK02013	*Shigella flexneri*	245	4,512,244	50.51	180	478	95,711	29,650	45	GCA_056643255.1	SRR39062443
DAK02014	*Shigella flexneri*	245	4,468,602	50.48	170	473	78,897	29,911	47	GCA_056643235.1	SRR39062439
DAK02015	*Shigella flexneri*	245	4,514,973	50.50	154	464	95,711	29,912	45	GCA_056643215.1	SRR39062438
DAK02018	*Shigella flexneri*	245	4,504,314	50.51	172	454	95,711	29,582	46	GCA_056643195.1	SRR39062437
DAK02022	*Shigella flexneri*	245	4,511,697	50.50	149	441	95,711	29,912	45	GCA_056643175.1	SRR39062436
DAK02023	*Shigella sonnei*	152	4,576,024	50.49	163	522	88,194	23,352	57	GCA_056643615.1	SRR39062435
DAK02024	*Shigella flexneri*	245	4,609,642	50.46	163	893	79,191	29,348	49	GCA_056643595.1	SRR39062434
DAK02031	*Shigella flexneri*	6,918	4,374,695	50.50	155	457	94,756	27,269	48	GCA_056643575.1	SRR39062433
DAK02034	*Shigella flexneri*	245	4,513,160	50.50	153	444	95,711	30,069	44	GCA_056643555.1	SRR39062453
DAK02059	*Shigella boydii*	145	4,376,029	50.63	168	499	107,787	20,574	63	GCA_056643535.1	SRR39062452
DAK02072	*Shigella sonnei*	152	4,705,658	50.53	141	566	88,196	23,963	58	GCA_056643515.1	SRR39062451
DAK02084	*Shigella flexneri*	245	4,528,028	50.45	119	464	95,640	29,650	46	GCA_056643475.1	SRR39062450
DAK03008	*Shigella flexneri*	145	4,472,541	50.73	160	590	90,346	19,991	66	GCA_056643495.1	SRR39062449
DAK03009	*Shigella flexneri*	245	4,461,913	50.48	160	456	79,191	30,827	46	GCA_056643455.1	SRR39062448
DAK03011	*Shigella sonnei*	152	4,601,768	50.50	161	525	88,194	23,963	57	GCA_056643415.1	SRR39062447
FAT07026	*Shigella flexneri*	245	5,544,605	50.25	134	1,972	95,656	22,394	67	GCA_056643435.1	SRR39062446
KAO05004	*Shigella sonnei*	152	4,583,510	50.49	159	550	88,199	23,232	59	GCA_056643395.1	SRR39062445
KMR16022	*Shigella sonnei*	152	4,554,467	50.51	147	545	88,197	23,353	58	GCA_056643375.1	SRR39062444
STL11028	*Shigella boydii*	243	4,343,724	50.63	163	598	69,921	17,545	72	GCA_056643355.1	SRR39062442
TAM13030	*Shigella boydii*	152	4,574,898	50.47	166	563	88,186	23,233	58	GCA_056643335.1	SRR39062441

The draft genome assemblies consisted of 752 to 2,396 contigs (≥500 bp), with N50 values ranging from 17,079 to 29,912 bp. Genome sizes ranged from 4.34 Mb to 5.54 Mb, with GC contents between 50.25% and 50.73%. All assemblies met quality thresholds, with completeness ≥90% and contamination <5%.

The data set included *Shigella flexneri* (*n* = 12), *Shigella sonnei* (*n* = 7), and *Shigella boydii* (*n* = 3), representing diverse strains circulating in Senegal.

## Data Availability

These Whole Genome Shotgun projects have been deposited in GenBank under BioProject PRJNA1376263. The corresponding raw sequencing reads are available in the NCBI Sequence Read Archive (SRA) under the same BioProject. Individual SRA run accession numbers (SRR) are listed in Table 1, together with the genome accession numbers.
